# De Winter’s pattern: an uncommon but very important electrocardiographic sign in the prompt recognition of spontaneous occlusive coronary artery dissection in young patients with chest pain (a case report)

**DOI:** 10.11604/pamj.2021.39.112.29512

**Published:** 2021-06-08

**Authors:** Nahid Azdaki, Marjan Farzad, Seyedali Moezi, Neda Partovi, Reza Ashabyamin

**Affiliations:** 1 Cardiovascular Diseases Research Center, Department of Cardiology, School of Medicine, Birjand University of Medical Sciences, Birjand, Iran,; 2 Razi Clinical Research Development Unit (RCRDU), Birjand University of Medical Sciences, Birjand, Iran,; 3 Cardiovascular Diseases Research Center, School of Nursing and Midwifery, Birjand University of Medical Sciences, Birjand, Iran,; 4 Forensic Medicine Research Center, Birjand, Iran

**Keywords:** Electrocardiogram, de Winter’s pattern, coronary artery dissection, case report

## Abstract

Chest pain is a clinical symptom for immediate consultation, and electrocardiogram (ECG) is a valuable diagnostic tool for use in the emergency room. Although the ST- elevation myocardial infarction (STEMI) requires urgent management, there are other ECG high-risk findings which are associated with adverse outcomes or imminent acute myocardial infarction (AMI). This is a case of STEMI equivalent pattern such as de Winter. As this ECG pattern is uncommon, it may be misinterpreted in the emergency department. We report a misinterpretation of de Winter's pattern (dWp) in a young woman referred to the emergency department for chest pain, feeling of suffocation and hemodynamic instability who undergone reteplase treatment with the suspicion of acute massive pulmonary embolism but developed cardiogenic shock as a result of extensive myocardial infarction due to spontaneous dissection of the left anterior descending coronary artery. A prompt diagnosis of de Winter's pattern and early angiography to detect the underlying cause of clinical manifestation can be lifesaving.

## Introduction

Recognition of electrocardiogram is a core clinical key in the emergency department medicine. A remarkable number of patients with acute myocardial infarction, caused by acute occlusion of an epicardial coronary artery, do not show ST-elevation on the electrocardiogram. This is the case of STEMI equivalent pattern which is characterized by loss of R waves in the precordial leads associated with up- sloping ST-segment depression at J-point > 1mm and tall positive symmetrical T waves [1]. This pattern of ECG is an infrequent but highly suggestive finding of the occlusion of the left anterior descending coronary artery that is vital to ensure timely reperfusion therapy or surgery [1, 2]. Misinterpretation of this risky pattern may lead to irreversible outcomes.

## Patient and observation

**Patient information:** a young and alert woman with no known cardiac history, referred to the emergency department by emergency medical service, presenting chest discomfort, acute dyspnea and feeling of suffocation.

**Clinical findings:** when examined, she had signs of shock: tachycardia (pulse 140/min), filiform pulse, sys BP < 90 mm/Hg and RR 20/min.

**Diagnostic assessment:** the 12-lead ECG was taken to screen the patient for possible cardiac ischemia. Her ECG did not show any acute ST elevation from the EMS specialist's point of view.

**Therapeutic intervention:** due to acute shortness of breath, hemodynamic instability and suspicion of acute massive pulmonary embolism, the EMS specialist recommends reteplase treatment 20unit/IV (two separate doses of 10 units 30 minutes apart) and concurrent cardiologist counseling.

**Figure 1:**
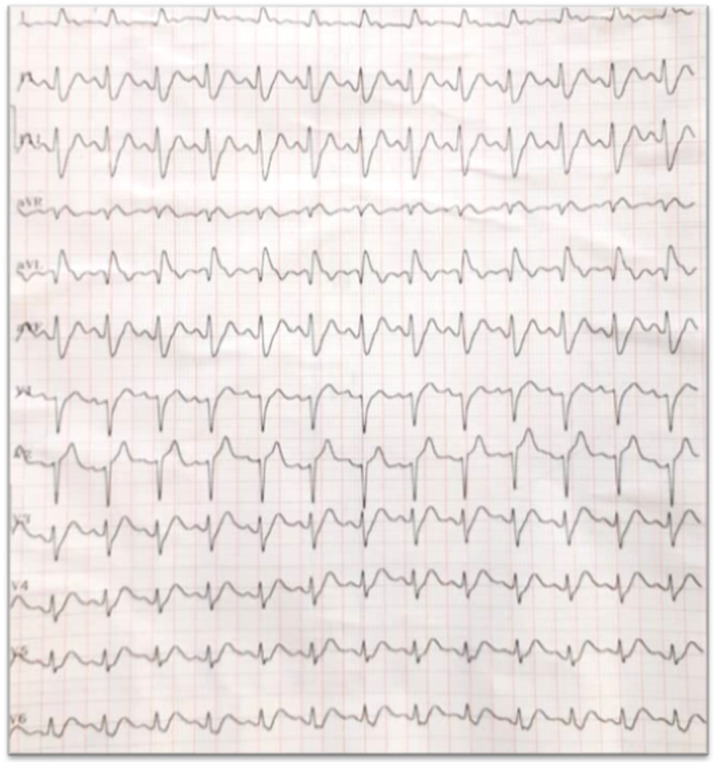
initial electrocardiogram: sinus tachycardia-NAD-up sloping ST-segment depression in inferior leads and V3-V6 and ST elevation in AVR, Normal QT: De winter type A

**Figure 2:**
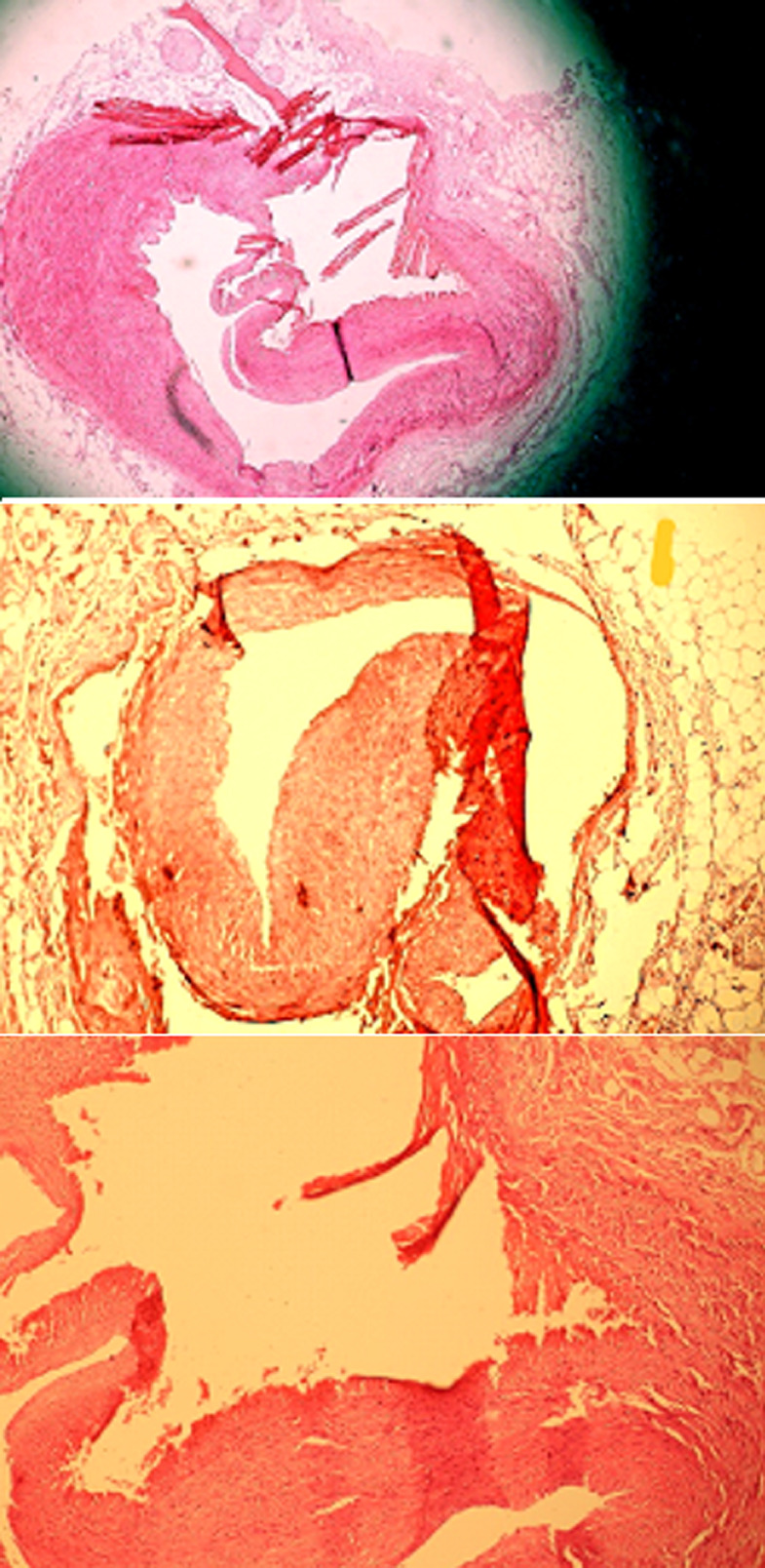
histopathological view of the left coronary artery: this is the left coronary artery from origin to the bifurcation site that, due to the sudden rupture and subsequently acute hemorrhage within the media layer, distended the wall and bulged the portion of inferior medial and internal layers into the lumen, caused luminal obstruction

**Follow-up and outcomes:** in a reanalysis by a cardiologist, the initial ECG was interpreted as ST elevation with the de Winter's pattern (Figure 1). After deteriorating of the patient's hemodynamic state and development of acute pulmonary edema with difficult breathing and bloody froth, emergency portable echocardiography was performed and showed abnormal ventricular wall motion in apex, lateral and septal, and severely reduced left ventricular ejection fraction (30%). Right ventricular and valve functions were both normal. Unfortunately, we couldn't perform urgent coronary angiography because of rapid deterioration of vital signs and asystole despite prolonged high quality cardiopulmonary resuscitation. The cause of death was determined to be extensive myocardial infarction due to spontaneous dissection of the ostio-proximal portion of the left anterior descending artery by forensic medicine two months later (Figure 2).

## Discussion

Acute coronary syndrome (ACS) diagnosis is based on clinical history, physical examination, electrocardiographic changes and biomarkers. One third of the patients, present ST-segment elevation in their first electrocardiogram (ECG) [3]. It is estimated that approximately 30% of ACS diagnoses are lost or delayed by misunderstanding of patterns warranting emergency coronary angiography such as STEMI equivalent [4]. Robert J de Winter described an ECG pattern that signifies the proximal LAD occlusion, it is evidenced by ST-depression from 1 to 3 mm in precordial leads V1-V4, with high and symmetrical T waves in the same leads, associated with ST elevation in AVR [4]. This pattern of ECG is an infrequent finding but its recognition is vital to ensure timely appropriate reperfusion therapy or surgery. Given, the potential morbidity and mortality resulting from failure to recognize these findings, knowledge of this pattern must be mandatory for all professionals involved in this network to reduce the morbidity and mortality complications of these high-risk patients.

Spontaneous occlusive coronary artery dissection (SCAD) is iatrogenic epicardial coronary artery dissection unrelated to an atherosclerotic disease process. SCAD is responsible for a small percentage (1%-4%) of acute coronary syndrome cases. The left anterior descending (LAD) artery is the most common artery affected in SCAD [5]. Myocardial injury in SCAD results from coronary artery obstruction secondary to intimal disruption. Clinical diagnosis of SCAD is difficult as there are no differentiated symptoms from the other cause of ACSs and its diagnosis is just based on angiography or pathology. Further, 70% of the cases are women, so diagnosis of SCAD should be strongly considered in a young woman with no pre-existing history of coronary artery disease who has chest pain [6] and ECG is a simple and valuable tool for the diagnosis of chest pain etiology in the emergency room, especially, in this group of patients who are young, female and have proximal involvement of great coronary arteries.

In this case, misinterpretation of the deceptive ECG and rapid deterioration of the patient accounted for the missing of a typical presentation of SCAD. Common conditions which predispose to SCAD include inherited arteriopathies, connective tissue disorders, exogenous hormone use, systemic inflammatory disease and coronary artery spasm. More than half of the patients remember a precipitating factor such as intense exercise, intense Valsalva maneuver, vomiting, intense emotional stress and drug abuse. Chest pain is the presenting symptom in the majority of the cases with most presenting as ACS [7-10]. Intense emotional stress is more likely to be a predisposing factor in our case; however, possible evidence of head trauma which has been reported by forensic medicine and its contribution to the patient's clinical status is a little questionable. Thrombolytic therapy should be avoided in SCAD as it leads to clinical deterioration due to the expansion of dissection. It is shown that 60% of patients who received thrombolysis have clinical deterioration requiring percutaneous coronary intervention (PCI) or coronary artery bypass grafting (CABG). Thus, early coronary angiography is vital if SCAD is suspected. However, in remote centers without transferring algorithms for primary PCI, thrombolysis should not be withheld for ST-elevation MI patients because of the higher frequency of thrombotic occlusion than SCAD.

## Conclusion

Our clinical case underlines the importance of recognizing de Winter's pattern to advance the patient to a rapid reperfusion strategy and confirm the high-risk and the probable evaluative feature of these ECG findings. The diagnosis of spontaneous occlusive coronary artery dissection should be considered in all young cases with chest pain and minimal traditional atherosclerotic risk factors referring to the emergency room. In this case, it is better to transfer these patients immediately to the cath lab for early invasive angiography and primary percutaneous coronary intervention.
